# Determination of vanillin in different food samples by using SMM/Au@ZIF-67 electrochemical sensor

**DOI:** 10.1038/s41598-023-45342-6

**Published:** 2023-10-20

**Authors:** Sara Dehdashtian, Shengnian Wang, Teresa A. Murray, Mahdieh Chegeni, Sadegh Rostamnia, Nazir Fattahi

**Affiliations:** 1 Institute for Micromanufacturing, Louisiana Tech University, Ruston, LA 71270 USA; 2 Center for Biomedical Engineering and Rehabilitation Sciences, Louisiana Tech University, PO Box 10157, Ruston, LA 71272 USA; 3 Department of Chemistry, Faculty of Science, Ayatollah Boroujerdi University, Boroujerd, 69199-69737 Iran; 4 Organic and Nano Group (ONG), Department of Chemistry, Iran University of Science and Technology (IUST), PO Box 16846-13114, Tehran, Iran; 5 Research Center for Environmental Determinants of Health (RCEDH), Health Institute, Kermanshah University of Medical Sciences, Kermanshah, Iran

**Keywords:** Chemistry, Electrochemistry

## Abstract

Vanillin is a popular flavoring agent in many food products. Simple, fast, and reliable quantification of this compound is crucial for the food industry. In this work, we have developed a new electrochemical sensor for accurate detection of vanillin in various real samples. The composite electrode was made of sodium montmorillonite nanoclay (SMM) and gold nanoparticles modified ZIF-67 (Au@ZIF-67), in which SMM contributes to the large adsorption capacity of the analyte, ZIF-67 and SMM supply more sensing active sites, and gold nanoparticles provide high electrical conductivity. The sensing electrode was comprehensively characterized using Brunauer–Emmett–Teller, EDS, XRD, SEM, FTIR, and TEM, and its electrochemical behavior for determination of vanillin including the electrooxidation mechanism of vanillin and different parameters such as scan rate and pH value was investigated. The result revealed that a two electron-two proton process was involved in the electrooxidation of vanillin, which takes place more readily due to the lower potential on the surface of SMM/Au@ZIF-67/carbon paste electrode. The new composite electrode was also more sensitive to vanillin detection with an anodic peak current almost 2.6 times more than that of the bare electrode. A linear sensing concentration range was established between 1 and 1200 nM with a detection limit of 0. 3 nM and a limit of quantitation of 1 nM. For real samples, the sensor demonstrated excellent recovery rates and reliability that was comparable to the standard high-performance liquid chromatography method.

## Introduction

As the main compound in natural vanilla, vanillin is a popular flavoring additive in many food, beverage, and pharmaceutical products^1^. Although well-known for its pleasant aroma and taste, vanillin may potentially benefit human health for its antioxidant, anti-inflammatory, and pain relief properties. For example, vanillin is found to be effective to reduce the risk of heart and liver diseases when consumed at moderate levels. But excessive use of vanillin could also cause negative side effects such as liver and kidney damage^2^. Therefore, it is important to monitor and regulate the daily consumption of vanillin in food (e.g., less than 10 mg/kg) based on the stipulation of The United Nations Food and Agriculture Organization^3^. Precise detection of vanillin requires high-performance liquid chromatography^4^ or capillary electrophoresis^5^, which are not only expensive but also time-consuming. Therefore, simple, fast, and reliable sensors are needed to monitor the quality and consistency of food products that contain vanillin.

Current sensors quantify the concentration of vanillin by measuring the changes in the electrical properties (electrochemical sensors) or color (colorimetric sensors) of a testing sample. Vanillin colorimetric sensors rely on the use of p-dimethylaminocinnamaldehyde, which reacts with vanillin to form a blue or purple complex, and the latter is then quantified using a spectrophotometer such as ultraviolet–visible spectrophotometry (UV–Vis) or a colorimeter ^6^. Electrochemical sensors for vanillin are designed to detect potential or current changes when vanillin molecules undergo an electrochemical reaction at the surface of the working electrode. Compared to the colorimetric sensing mechanism, electrochemical vanillin sensors have advantages such as low-cost, fast response, and high sensitivity and selectivity. As the critical component of electrochemical sensors, the material and architectures of the working electrode such as large surface area and high electrical conductivity determine the overall electrochemical performance during vanillin sensing.

The novel nanocomposites have been used successively for fabrication of developed electrochemical sensors^7–11^. The outstanding properties of nanocomposite modified electrodes such as the increased sensitivity and selectivity make them the ideal candidates for detection of different chemical and biological compounds. Therefore, we have used a novel nanocomposite (SMM/Au@ZIF-67) for designing an electrochemical sensor to detect vanillin in different real samples.

In this work, we used sodium montmorillonite nanoclay and gold nanoparticle modified ZIF-67 to fabricate the electrode for electrochemical sensing vanillin.

The main advantage of using of sodium montmorillonite nanoclay for determination of vanillin is accumulation of vanillin at the surface of the modified electrode by SMM due to positive charge of this compound at pH = 7. Vanillin has an ionizable functional group (hydroxyl) with a pKa value of 7.4. Therefore, at pH = 7, vanillin exists as a cation (due to the hydroxyl moiety with positive charge) and their electrostatic interactions with the negatively charged SMM allow vanillin to accumulate at the surface of the modified electrode by SMM which leads to the sensitivity increase of the modified electrode by SMM.

On the other hand, Metal–organic frameworks (MOFs) have been applied as another key compounds in this electrode material because of their large surface area, rich metal sites and high porosity. The obtained properties are based on the selection of organic ligands, and metal sites^12^. The MOFs are preferred as an active electrode material for dual applications such as supercapacitor and sensors due to their multiple valence state, cost-effectiveness, and stable structure^13^.

MOF materials were reported to effectively catalyze reactions and enhance electron transfer rate in electrochemical reactions^14^.

Gold nanostructures are the most investigated metal nanostructures in analytical chemistry owing to their extraordinary electrocatalytic, electronic and optical properties^15^. The presence of gold nanoparticles provide excellent conductivity, chemical stability, and further surface-to-volume ratio to further leverage the sensitivity of an electrochemical sensor^16^. The In addition, the layered structure of the SMM nanoclays helps absorb the target compounds effectively^17^. Despite the advantages of each component in this composite electrode having been verified separately by us or by other research teams, this work evaluates the electrochemical sensing performance of the new composite electrode. The electrooxidation of vanillin was investigated and compared with the bare electrode counterparts both in prepared samples and real food products and the overall sensing performance was further validated by the standard HPLC method.

By using the extraordinary properties of SMM and Au@ZIF-67, which include increasing surface area and conductivity, we strongly believe that the SMM/Au@ZIF-67-based sensor is an ideal sensor for highly sensitive and selective detection of vanillin in different real samples.

Also, the sensor has desirable characteristics such as fast operation, reproducibility, accuracy, low-cost, negligible sample, and solvent consumption. Furthermore, it is environmentally friendly. To the best of our knowledge, the proposed sensor is a novel electrochemical sensor and there is not any another report of using SMM/Au@ZIF-67 composite for fabrication of electrochemical sensor.

## Experimental

### Materials and instruments

The graphite powder was purchased from Merck and used for fabrication of carbon paste electrodes. Sodium phosphate salts were purchased from Merck and used in the preparation of phosphate buffer solution in electrochemical experiments.

All other reagents were of analytical grade and were purchased from Sigma-Aldrich.

### Synthesis of metal–organic framework ZIF-67

ZIF-67 was synthesized according to our previous procedures in which 0.225 g (0.77 mmol) Co(NO_3_)_2_.6H_2_O was dissolved in 12 mL deionized water and 0.622 g (0.76 mmol) 2-methylimidazole (C_4_H_6_N_2_) was dissolved in 80 mL deionized water. The two solutions were then mixed under vigorous agitation for 6 h. After keeping the solution in a stainless-steel autoclave at 60 °C for 24 h, a purple precipitate was collected by centrifuging three times using methanol as the eluent. After drying, the precipitate was designated as as-prepared ZIF-67^18^.

### Synthesis of the Au nanoparticles modified metal–organic framework (Au@ZIF-67)

**Figure 1:**
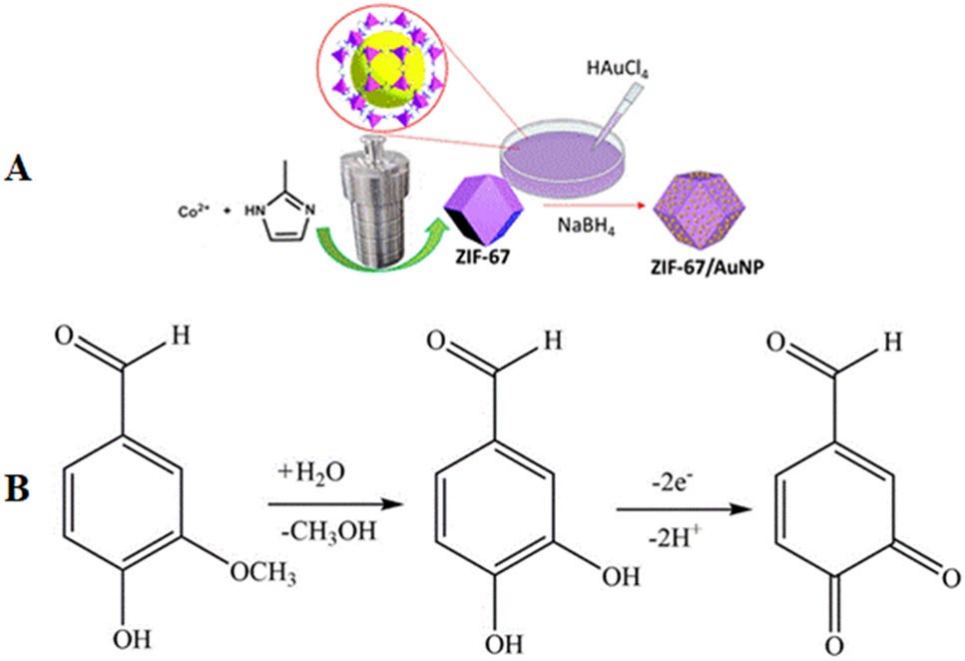
(**A**) The multi-step synthesis of Au@ZIF-67 composites, and (**B**) The electrooxidation mechanism of vanillin at the surface of SMM/Au@ZIF-67/CPE.

To load gold into the nanopores of ZIF-67, a wet impregnation method was utilized. First, 0.2 g MOF produced from the previous step was spread on a watch glass by a glass rod. Then, 1.6 mL of a HAuCl4 aqueous solution (2 wt% Au) was added dropwise while stirring with a glass rod until it dried. The ZIF-67/Au solid was then loaded in a water/DMF solution (1:1 10 mL) in which sodium borohydride (1 M) was pre-added. In this way, gold ions were loaded on the surface of MOF and further reduced in situ by NaBH_4_. After 30 min, the mixture was centrifuged, and the solid was washed with DMF and ethanol, and dried in an oven at 60 °C. The synthesis process of Au@ZIF-67 is shown in Fig. 1A.

### Preparation of electrochemical sensor

Both the new composite electrode and benchmark bare electrode were tested for vanillin sensing. The carbon paste of the bare working electrode was made of graphite powder and paraffin oil (as the binder) using the same procedure previously reported by our group^19,20^. For the new composite working electrode (as the final electrochemical sensors), in addition to graphite powder and paraffin oil, the optimized amounts of SMM and Au@ZIF-67 were incorporated into the body of the electodes.

### Analytical procedure

For voltametric experiments, a 10 ml phosphate buffer solution was used in an electrochemical cell. The potential scan window was set from 0.0 to 1.2 V for cyclic voltammetry (CV) and differential pulse voltammetry (DPV). For DPV, the optimized parameters were 50 mV pulse amplitude, 40 ms pulse width, and 60 mVs^−1^ scan rate. For cyclic voltammetry, a scan rate of 100 mVs^−1^ was used for recording of voltammograms.

In electrochemical investigations, an Autolab potentiostat (PGSTAT302N, Metrohm Autolab, Netherlands) was used with a 3-electrode configuration in which a platinum wire and saturated calomel electrode were utilized as the counter and reference electrodes, respectively. In this configuration, the carbon paste electrode (unmodified or modified) was used as the working electrode.

### Preparation of real sample

For real sample tests, 1 g of food sample (three brands of cake and biscuit) was dispersed in 10 mL ethanol with using ultrasonication for 30 min to obtain a homogeneous suspension. After centrifugation, the supernatant (~ 1.0 mL) was further diluted to 10 mL with 0.10 M PBS (pH = 7.0) and then used for further experiments.

The accuracy of electrochemical detection of vanillin was validated by HPLC (Knauer with Chromgate software version 3.1). The HPLC instrument was equipped with binary pumps (Smartline-1000–1 and Smartline-1000–2, Berlin, Germany), and a programmable detector (Smartline-UV-2500) for variable wavelengths, an on-line solvent vacuum degasser, and manual sample injector fitted with a 20 µL injection loop (Model 7725i, Rheodyne, Cotati, CA, USA). Separations were carried out on a H5-ODS C18 column (15 cm × 4.6 mm, with 5 µm particle size) from Anachem (Luton, UK). The mobile phase was a mixture of methanol buffer of 0.1% formic acid (45:55, v/v) and the flowrate was set at 1.0 mL min^−1^ in isocratic elution mode and the detection was performed at the wavelength of 280 nm.

## Results and discussion

### Characterization of Au nanoparticles modified metal–organic framework Au@ZIF-67

First, we characterized Au nanoparticle-modified MOF Au@ZIF-67 using different techniques including Fourier-transform infrared spectroscopy (FTIR), Brunauer–Emmett–Teller (BET) analysis, energy dispersive x-ray spectroscopy (EDS), x-ray diffraction (XRD), scanning electron microscopy (SEM) and transmission electron microscopy (TEM). These characterizations provided a more comprehensive understanding of MOF Au@ZIF-67 and the expected qualities of this modifier.

The FTIR spectra of the synthesized structures ZIF-67 and Au@ZIF-67 are reported in Figure S1 (See Supporting Information). The peaks at 1633 and 1416 cm^−1^ are related to the tensile vibration of the C=N and C=C groups of imidazole, respectively. Also, the peak of 3439 cm^−1^ is related to the tensile vibration of hydroxyl groups (O–H) and the peak at about 425 cm^−1^ is related to the stretch of the Co–N bond. After the adsorption process, due to the decrease in the electron density of energy of cobalt, the peak intensity in 425 cm^−1^ decreases. This phenomenon occurs when nitrogen in the ligand structure donates electrons to the cobalt ion.

X-ray powder diffraction (XRD) was performed to obtain the crystal construction of ZIF-67 and Au@ZIF-67 samples (Figure S1B). The XRD pattern of ZIF-67 is assigned by the peaks at 2θ = 7.42°, 10.46°, 12.81°, 14.79°, 18.23°, and 22.50°, which are corresponded to diffraction peaks at (011), (002), (112), (013), (222), and (114) crystal plane. Au@ZIF-67 XRD pattern is shown a clear peak of Au (111) at 2θ = 38.1°, and the crystal structure of metal–organic framework is relieved no clear change. The crystallinity of the ZIF-67 structure indicates no significant decrease in Au@ZIF-67, which indicates that the gold metal impregnated in the cavities of ZIF-67.

**Figure 2:**
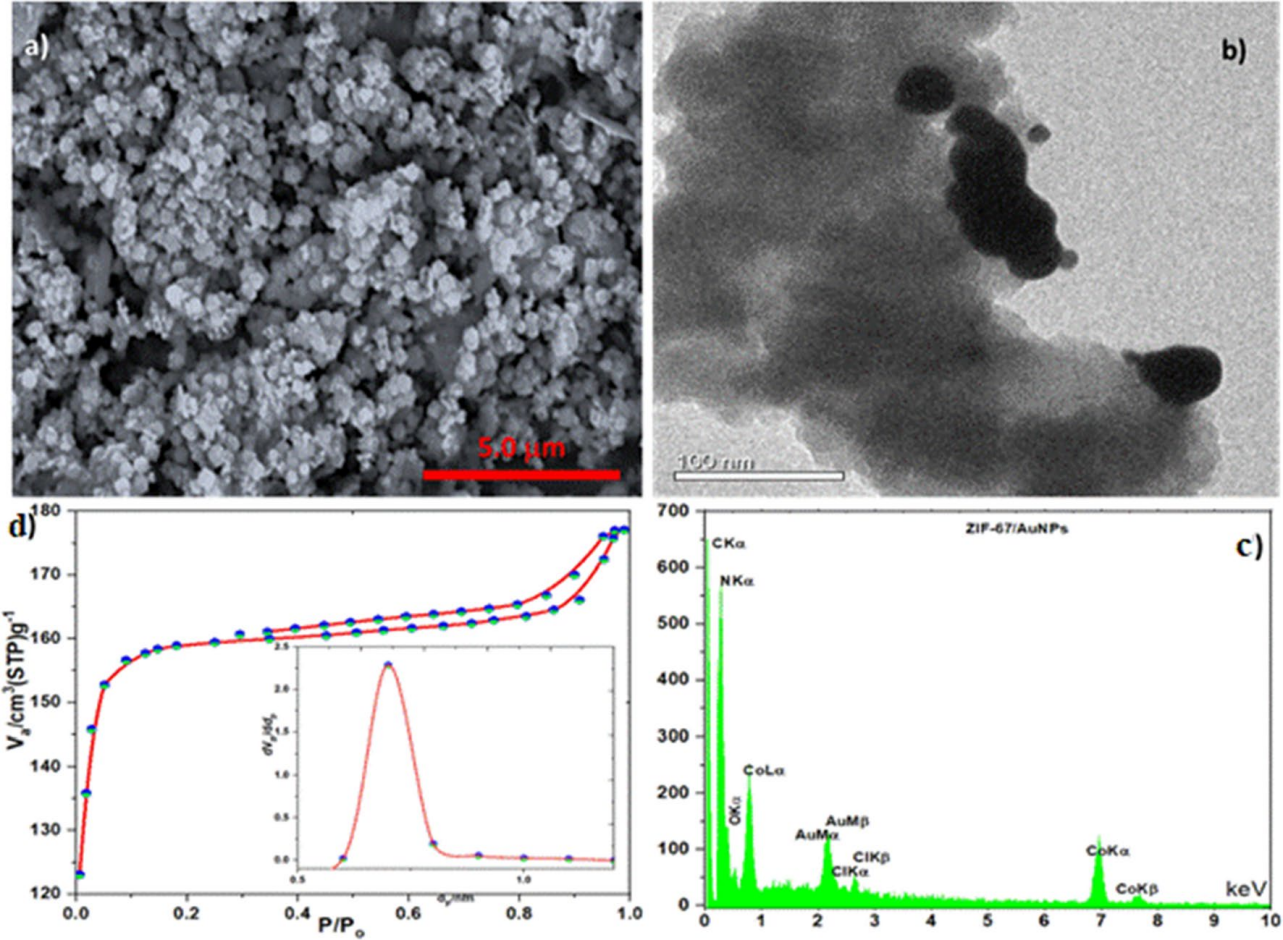
Au@ZIF-67: (**a**) SEM image, (**b**) TEM image, (**c**) EDS, and (**d**) BET (inset BJH).

For an additional investigation of the morphology and composition of Au@ZIF-67, SEM, TEM, and EDS analysis was done. The SEM image (Fig. 2a) shows that the grain size of the Au@ZIF-67 composites is ~ 300 nm and the TEM image (Fig. 2b) further reveals that gold nanoparticles are attached on the ZIF-67 structure. The EDS analysis of Au@ZIF-67 confirms the existence of Co and C atoms from ZIF-67 besides the presence of N and Au atoms in its structure (Fig. 2c). Given the importance of the surface area of the working electrode, the BET measurement of the Au@ZIF-67 was also carried out using N_2_ physical adsorption experiments (Fig. 2d). The N_2_ adsorption–desorption isotherms of Au@ZIF-67 shows a combination of both type I and type IV adsorption/desorption profiles with two uptake steps, a steep one at P/P0 < 0.02 followed by a slow one afterward. The first one is attributed to the filling of micropores of ZIF-67 while the second one suggests the existence of mesoporous space that is created by nanoparticle aggregates (Fig. 2d). A hysteresis loop at 0.40 < P/P0 < 0.95 further confirms the existence of large porous structures (which causes capillary condensation). The BET surface area of Au@ZIF-67 is calculated to be 324 m^2^g^−1^, and the total pore volume of Au@ZIF-67 is 0.302 cm^3^/g. The BJH pore size distribution of Au@ZIF-67 is calculated as 0.76 nm (inset of Fig. 2d), indicating the dominance of micropores due to ZIF-67.

### Characterization of composite SMM/Au@ZIF-67

**Figure 3:**
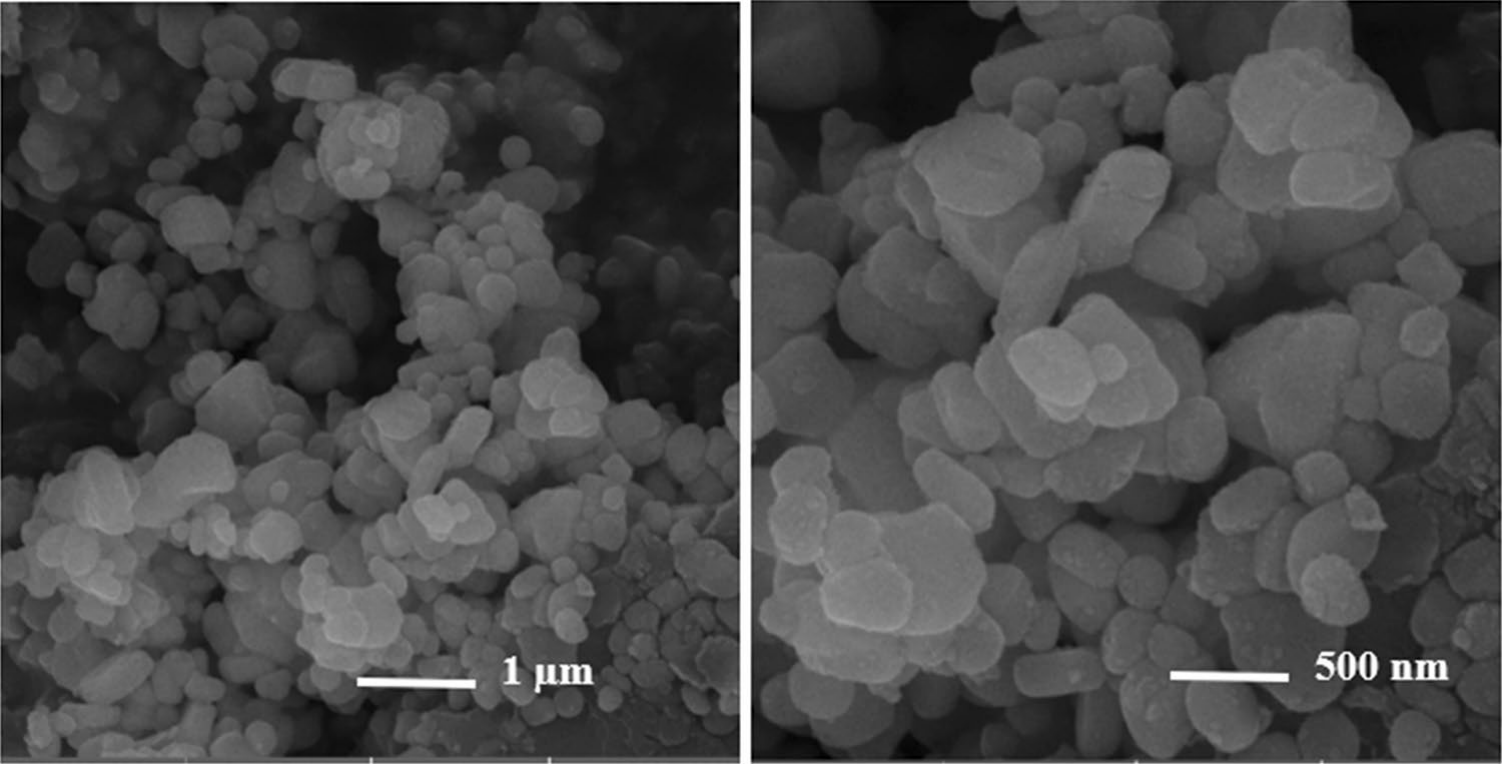
FESEM images of SMM/Au@ZIF-67.

The morphology of SMM/Au@ZIF-67 was determined by FESEM technique, as seen in Fig. 3. The SMM/Au@ZIF-67 images (two different magnificence) shows the construction of as-synthesized composite, which SMM sheets is integrated in Au@ZIF-67 as well.

### Characterization of the different CPEs

**Figure 4:**
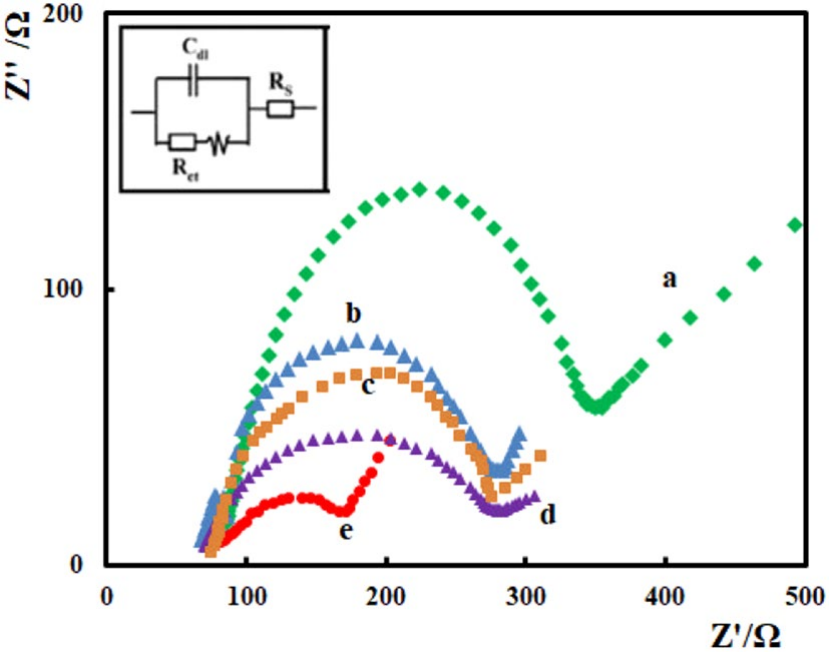
EIS for (**a**) bare CPE, (**b**) SMM/CPE, (**c**) Au@ZIF-67/CPE, (**d**) SMM/ZIF-67/CPE, and (**e**) SMM/Au@ZIF-67/CPE in 3 mM [Fe(CN)6] 3-/4- with 0.1 M KCl.

The CPEs were characterized by electrochemical impedance spectroscopy using [Fe(CN)6] ^3−/4−^ as the electrochemical redox probe. In electrochemical impedance measurement, the semicircle diameter of impedance equals the electron transfer resistance (R_et_), which controls the electron transfer kinetics of the redox probe at the electrode surface. Figure 4 presents the Nyquist diagrams of the different carbon paste electrodes in 3 mM [Fe(CN)6] ^3−/4−^ and 0.1 M KCl. SMM/Au@ZIF-67/CPE has a smaller semicircle at the high frequency region when compared with the other electrodes, indicating lower electron transfer resistance. This can be attributed to the presence of SMM, gold nanoparticles and ZIF-67 with good conductivity and large surface area in the modified electrode. This could effectively increase the rate of electron transfer between the electrode surface and [Fe(CN)6] ^3−/4−^ and decrease interface electron transfer resistance.

### Investigation of the electrochemical behaviors of vanillin at the surface of SMM/Au@ZIF-67/CPE

**Figure 5:**
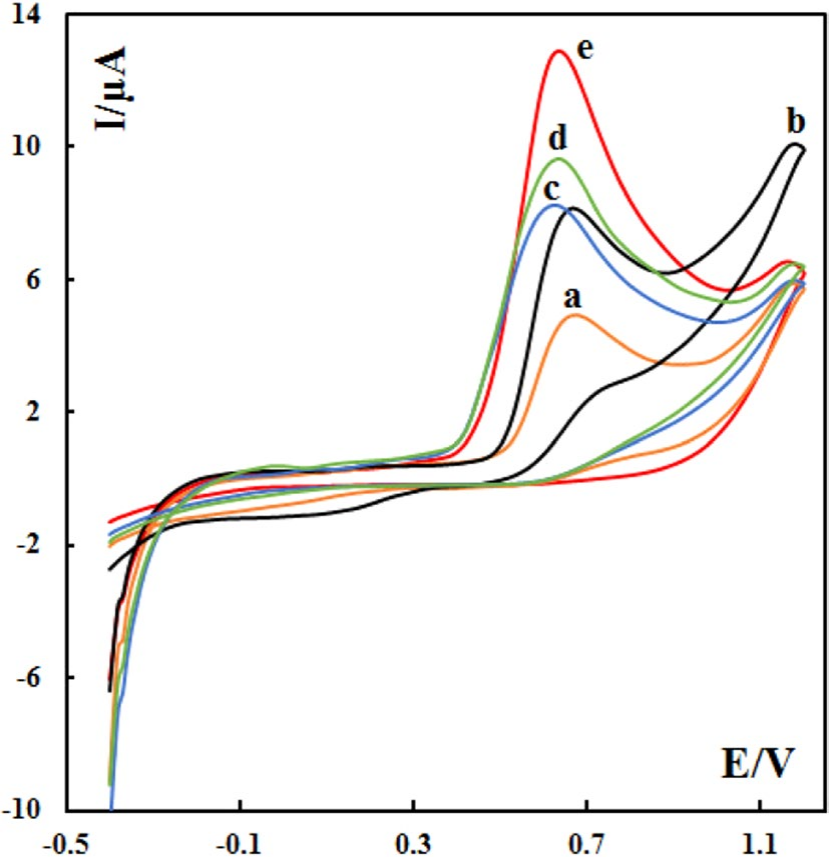
Cyclic voltammograms of different electrodes at 100 mVs^−1^ in phosphate buffer solutions (pH 7) in the presence of 400 nM vanillin: (**a**) bare CPE, (**b**) SMM/CPE, (**c**) Au@ZIF-67/CPE, (**d**) SMM/ZIF-67/CPE, and (**e**) SMM/Au@ZIF-67/CPE.

The electrochemical behavior of vanillin at the surface of SMM/Au@ZIF-67/CPE, SMM/ZIF-67/CPE, Au@ZIF-67/CPE, SMM/CPE, and bare CPE in phosphate buffer solution (pH 7) was investigated (Fig. 5). An anodic peak which is related to the electrooxidation of vanillin (Fig. 1B) was observed on all these electrodes. The lack of any cathodic peak in the reverse scan shows that the process is completely irreversible under these conditions. For bare CPE, the oxidation of vanillin shows a week anodic peak (E = 0.67 V and Ip = 4.93 µA). At the surface of SMM/CPE, the current of the anodic peak is higher. This shows that the layered structure of SMM nanoclay can increase both the surface area of the electrode and the transport space to allow vanillin molecules to diffuse into the coating so that more of them could adsorb at the surface of the working electrode and consequently the sensitivity of the electrode can be improved.

The main reason for adsorption of vanillin at the surface of SMM/CPE is the positive charge of this compound at pH = 7. Vanillin has an ionizable functional group (hydroxyl) with a pKa value of 7.4. Therefore, at pH = 7, vanillin exists as a cation (due to the hydroxyl moiety with positive charge) and their electrostatic interactions with the negatively charged SMM allow vanillin to accumulate at the surface of the SMM/CPE which leads to the sensitivity increase of the SMM/CPE. Similarly, at the surface of Au@ZIF-67/CPE, the current of the anodic peak also increased (Fig. 5, Curve c). Indeed, at the surface of this electrode, the gold nanoparticles and the metal organic framework help improve the electrical conductivity and the surface area, respectively. These two factors lead to a higher sensitivity of Au@ZIF-67/CPE than the bare electrode toward vanillin. In addition, the oxidation peak of vanillin shifts to a low potential position on the Au@ZIF-67/CPE (around 0.05 V). For another combination of the composite electrode, SMM/ZIF-67/CPE, a higher current of the anodic peak can be seen (Curve d). The most sophisticated electrode, SMM/Au@ZIF-67, shows the most remarkable increase in the sensitivity of vanillin and a similar negative shift at the oxidation peak (curve e). This is achieved with combined benefits from all three components in this SMM/Au@ZIF-67/CPE: more accessibility and adsorption of analyte (SMM), larger surface area (MOF and SMM), and higher electrical conductivity (gold nanoparticles).

### Investigation of the effect of pH on the electrochemical behavior of vanillin

**Figure 6:**
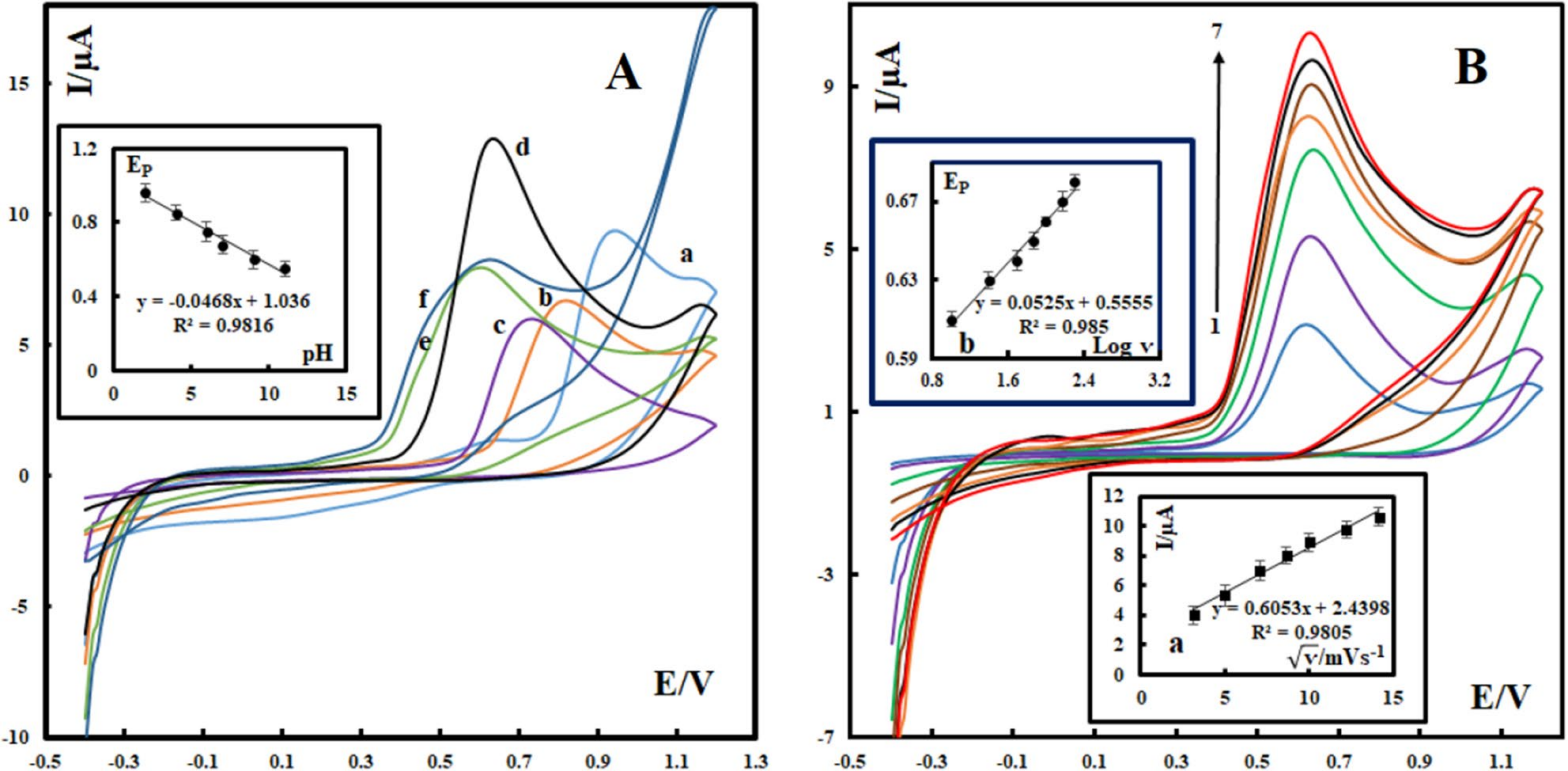
Cyclic voltammograms of SMM/Au@ZIF-67/CPE in phosphate buffer solutions (**A**) Effect of pH of PBS containing 400 nM vanillin with a constant scan rate of 100 mVs^−1^. The letters of a–f correspond to pH of 2, 4, 6, 7, 9 and 11 respectively. (**B**) Effect of the CV scan rate in PBS (pH 7) containing 280 nM vanillin. The numbers of 1–7 correspond to 10, 25, 50, 75, 100, 150 and 200 mVs^−1^ respectively. Inset a: variation of Ip with ʋ^1/2^. Inset b: variation of Ep versus log ʋ.

To achieve the highest sensitivity to vanillin, the optimized pH condition of this sensor was investigated. The cyclic voltammetry experiments were done in a pH range of 2 to 11 in phosphate buffer solutions that contained 400 nM vanillin. As shown in Fig. 6A, the maximum peak current was obtained at pH 7 and this was adopted for later sensing tests. Besides the dependence of sensitivity on pH, our investigations under various pH conditions also helped to understand the electrooxidation mechanism of vanillin at the surface of the electrode. As shown in Fig. 6A, with increasing pH value of the testing solution, the anodic peak potential of vanillin shifts towardsless positive values. Further analysis found a nearly linear relationship between the anodic peak potential and the pH value of the solution (inset of Fig. 6A), with a linear regression equation given by:

$${\mathrm{E}}_{\mathrm{pa}}\left(\mathrm{V}\right)=-0.0468\mathrm{ pH}+1.036\,({\mathrm{r}}^{2}=0.9816)$$

The slope (− 46.8 mV/pH) is close to the theoretical value of slope in Nernst equation, suggesting the participation of equal number of protons and electrons in the oxidation of vanillin on the modified CPE^20^.

### Investigation of the effect of the scan rate on the electrochemical behavior of vanillin

To further understand the electrooxidation process of vanillin at the surface of SMM/Au@ZIF-67/CPE, the effect of the CV scan rate on the peak current of vanillin was investigated. For this purpose, the cyclic voltammograms of 280 nM vanillin at different scan rates including 10, 25, 50, 75, 100, 150 and 200 mVs^−1^ were conducted (Fig. 6B). A linear relationship (y = 0.6053ʋ^1/2^ + 2.4398 (I: µA, ʋ: mVs^−1^)) with a correlation coefficient of R^2^ = 0.9805 was obtained between the peak current and the square root of the scan rate in this scan range (i.e., 10 to 200mVs^−1^). This revealed that the oxidation of vanillin is a diffusion-controlled process rather than an adsorption-controlled one in this range of scan rates. The relationship between the oxidation peak potential and logarithm of the scan rate was established as follows: E_pa_ = 0.0525 log ʋ + 0.5555, r = 0.985. According to Laviron’s theory^21^, the slope was equal to 2.303*RT*/ α*n*_*α*_*F*, where α, *n*_*α*_ , F, R and T are the electron transfer coefficient, the number of transferred electrons, the Faraday constant, the universal gas constant and the temperature in Kelvin, respectively. The value of α*n*_*α*_ was found to be 1.1. Thus, the rate-limiting step is close to a two-electron transfer process since the transfer coefficient (α) is assumed to be 0.5 (thus the value of *n*_*α*_ is approximately 2). Since equal numbers of electrons and protons take part in the oxidation of vanillin, the transfer of two electrons and two protons must be involved in the electrochemical reaction. These observations confirm the proposed oxidation process presented in Fig. 1B. The same mechanism for electrooxidation of vanillin has been previously reported ^22–24^. Since the chemical structure of vanillin is like a substituted catechol, the observation of a two electron-two proton process is expected.

### Analytical performance

**Figure 7:**
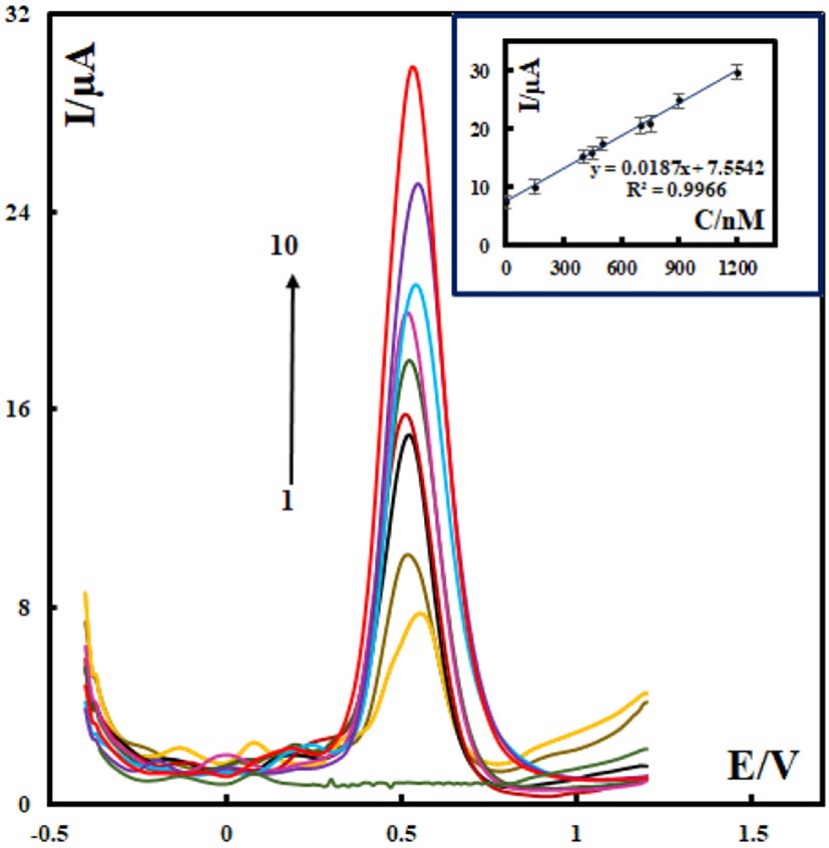
DPV results of SMM/Au@ZIF-67/CPE of vanillin in phosphate buffer solutions (pH 7). Insets show the plots of the peak current as a function of vanillin concentration in the range of 0, 1, 150, 400, 450, 500, 700, 750, 900 and 1200 nM.

Differential pulse voltammetry (DPV) is one of the most sensitive electrochemical methods. The anodic current of vanillin of SMM/Au@ZIF-67/CPE in DPV was measured in samples that have different concentrations of vanillin, and the results were shown in Fig. 7. A calibration curve of the DPV current versus the vanillin concentration was plotted within the range 1–1200 nM (the inset of Fig. 7), and a linear regression equation was found as follows:

$${I}_{P}\left(\mu A\right)=7.5542+0.0187{C}_{vanillin (nM)} ({r}^{2}=0.9966) A$$

Based on these observations, the limit of detection (LOD) and the limit of quantitation (LOQ) of SMM/Au@ZIF-67/CPE for sensing vanillin are 0.3 nM and 1 nM respectively.

The sensitivity (S) of a sensor is defined as the gradient of the linear portion of the curve, as shown in Eq. 1.

1 $$ {\text{S }} = \, \Delta {\text{I}}/ \, \Delta {\text{C}} $$

The LOD is defined as the lowest concentration of analyte that can be reproducibly determined, with a specified level of confidence, in a sample compared to a blank measurement. The LOD can be calculated using Eq. 2.

2 $$ {\text{LOD }} = {\text{ k }} \times \, \sigma \, /{\text{ S}} $$

where σ is the standard deviation of the blank measurements (i.e., noise level), S is the sensitivity calculated using Eq. 1, and k is the confidence level parameter. The confidence level parameter is set as k = 3 in the determination of the LOD. Alternatively, the confidence level parameter is set as k = 10 in the determination of the level of quantification (LOQ)^25^.

The detection limit for this work is lower than previously reported values of electrochemical sensors such as those that used NiFe2O4/1H3MCl/CPE^26^, C3N4/GCE^3^, MWNTs-PDA@MIP/SWNTs-COOH/GCE^27^, FePc MOF/GCE^22^, f-MWCNTs-FNTs/CPE^28^, MnO2 NWs-rGO/GCE^29^, MoS_2_-CNF/GCE^1^, CuO@SiO_2_-modified electrode^2^ and LCO@CNF/RDE^30^ (Table 1). Our new SMM/Au@ZIF-67/CPE exhibits more sensitive detection of vanillin when compared to those in the literature.

**Table 1:** Comparison of the performance of various electrodes for vanillin detection.

Electrode	Method	Linear range	Detection limit	References
NiFe_2_O_4_/1H3MCl/CPE	DPV	0.003–400.0 μM	1.0 nM	Zabihpour et al. (2020)
C_3_N_4_/GCE	DPV	20 nM to 10 μM and 15–200 μM	4 nM	Fu et al. (2020)
MWNTs-PDA@MIP/SWNTs-COOH/GCE	DPV	0.2–10 μM	0.1 μM	Wu et al. (2017)
FePc MOF/GCE	DPV	0.22–29.14 µM	0.05 µM	Peng et al. (2020)
f-MWCNTs-FNTs/ CPE	CV	5 × 10^−8^ to 9 × 10^–6^ M (trace levels) and 10^–5^ to 10^–4^ M (higher concentrations)	3.4 × 10^−^8 M	Taouri et al. (2021)
MnO_2_ NWs-rGO/GCE	SDLSV	0.01–20 µM and 20–100 µM	6.0 nM	Tian et al. (2020)
MoS_2_-CNF/GCE	Amperometry	0.3 to 135 μM	0.15 μM	Qianwen et al. (2019)
CuO@SiO_2_-modified electrode	DPV	0.05–1.2 μM and 6.2–111.2 μM	53 nM	Venkadesh et al. (2021)
LCO@CNF/RDE	Amperometry	0.01–1670 μM	4.67 nM	Wang et al. (2023)
SMM/Au@ZIF-67/CPE	DPV	1–1200 nM	0.3 nM	This work

Also, the proposed electrochemical sensor has desirable characteristics such as fast operation, reproducibility, accuracy, low-cost, negligible sample and solvent consumption, and environmentally friendly. Having these properties makes this sensor a powerful tool for detection of vanillin in real samples.

Based on all observations for this work, there are not any major limitations (disadvantages) for electrochemical determination of vanillin by using SMM/Au@ZIF-67/CPE.

Repeatability is another important criterion when assessing the performance of an electrochemical sensor. For this assessment, 10 replicate measurements (200 nM vanillin) were acquired, and the recovery and the relative standard deviation (RSD) was above 98% and lower than 3.4%, respectively. Based on these findings, this new electrochemical sensor can give reliable performance for real samples.

The reproducibility of the electrode was investigated by using DPV. Three freshly packed electrodes were prepared on three consecutive days and the peak current values of a solution containing 200 nM vanillin were measured for each electrode. The results from five replicates had a standard deviation of less than ± 0.96, suggesting that the probe has desirable reproducibility. The longterm stability of the modified carbon paste electrode was also evaluated. The electrode retained 95% of its initial activity after 3 weeks when stored in at room temperature, demonstrating high stability.

### Interference study

To investigate the potential for interference from non-target ions and molecules during vanillin sensing, F^−^, Li^+^, Na^+^, Br^−^, Ca^2+^, Al^+3^, Zn^2+^, Glucose, p-hydroxybenzoic acid, phenylalanine, glycine, vitamin B_2_, vitamin B9, L-tyrosine, guaiacol and ethylvanillin were added to samples with vanillin (400 nM) and analyzed using DPV. Our findings show that a 500-fold concentration increase of these compounds had no meaningful effect on the current at this low concentration of vanillin, with an acceptable tolerance of $\pm$ 5% of the DPV current. These mentioned ions/molecules did not interfere with the oxidation peak of vanillin in this condition. Based on these observations, the fabricated electrochemical sensor can be considered as a highly selective sensor toward vanillin.

### Analytical application

For DPV investigation of real food samples, the testing solutions were prepared with a procedure described in Sect. “Preparation of real sample” and the vanillin concentration was further verified with a standard HPLC analysis. For the HPLC method, about 1.0 g of each sample was dissolved in 100 mL ultra-pure water and acetonitrile (50:50 v/v). After sonicating for 30 min in an ultrasonic bath, the testing solution was filtered with a syringe filter (0.45 μm) and was subjected to HPLC–UV analysis.

The measurement results for DPV (Table 2) showed that the content of vanillin was 20 nM or 0.3 µg/g^−1^ in Ashena Cake, 23 nM or 0.34 µg/g^−1^ in Lahijan Cake, and 11.5 nM or 0.167 µg/g^−1^ in Baby Biscuits. The corresponding vanillin concentration of these samples measured by HPLC were 19.5 nM or 0.296 µg/g^−1^ in Ashena Cake, 22.51 nM or 0.342 µg/g^−1^ in Lahijan Cake, and 12.17 nM or 0.185 µg/g^−1^ in Baby Biscuit. The related chromatograms are shown in Supplemental Figure S2.

**Table 2:** Determination of vanillin in real samples using DPV (n = 4).

Samples	Added (nM)	Found (nM)	Recovery (%)	RSD (%)
Sample 1 (Ashena cake)	0	20	–	3.1
	10	31	103	3.5
	20	42	105	3.8
	40	58	96	2.6
Sample 2 (Lahijan cake)	0	23	–	2.7
	10	32	96	3.5
	20	44	102	2.6
	40	62	98	2.8
Sample 3 (Baby biscuit)	0	11.5	–	3.4
	10	22	102	2.1
	20	33	104	2.6
	40	50	97	2.9

The differences between the results were checked by the F-test method, and the calculated F-values for the related samples were 1.81, 2.30 and 1.84 for Athena Cake, Lahijan Cake, and Baby Biscuits, respectively. In all cases, the calculated F-values were lower than the F-value of 6.39 expected at *P* = 0.05, indicating that there was no statistically significant difference between the values obtained by two methods. Also, the results for t-test are 0.26, 0.2 and 0.57 respectively. All these values are lower than critical t for degree of freedom 6 (2.45). Thus, these results confirm the reliability of the proposed sensor.

These results show that the DPV method with our SMM/Au@ZIF-67/CPE sensor measures the concentration of vanillin as well as HPLC. Also, the observed results for the standard addition of analyte in DPV method can be seen in Figure S3 and Table 2.

## Conclusions

In this work, we developed a highly sensitive and selective electrochemical sensor for the detection of vanillin. The sensing composite electrode was made by adding SMM and Au@ZIF-67 in carbon paste which provided superior performance due to larger adsorption capacity of analyte contributed by SMM, more surface area by MOF and SMM, and higher electrical conductivity by gold nanoparticles. This new composite electrode was found to be more sensitive for the determination of vanillin concentration in comparison to bare electrode with an observed peak current almost 2.6 times higher and easier occurrence of the electrooxidation of vanillin with its lower potentials. The electrooxidation of vanillin on the surface of SMM/Au@ZIF-67/CPE was investigated at various pH values, scan rates, and in the presence of interfering ions and molecules. A LOD of 0.3 nM and a LOQ of 1 nM were achieved for this new SMM/Au@ZIF-67/CPE, which is better than many other sensors in the literature. The sensor shows a recovery of 98 + % and an RSD of lower than 3.4% with an acceptable tolerance of $\pm$ 5% for signal repeatability. As for vanillin sensing in real samples, including baby biscuits and cakes that are commonly flavored with vanillin, this electrochemical sensor achieved 96–105% of the recovery rates that were verified by HPLC. These results show that our new sensors can be used successfully in the detection of vanillin in real samples with high precision.

### Supplementary Information

Supplementary Figures.

## Data Availability

All data generated or analysed during this study are included in this published article (and its Supplementary Information files).
