# Supplementary material for: Determination of vanillin in different food samples by using SMM/Au@ZIF-67 electrochemical sensor
Source: Sci Rep. 2023 Oct 20;13:17907. doi: 10.1038/s41598-023-45342-6 (PMC10589296; doi:10.1038/s41598-023-45342-6)
Supplement: Supplementary file 1 — Supplementary Figures. [file 41598_2023_45342_MOESM1_ESM.docx]

**Supporting information**

**Determination of vanillin in different food samples by using SMM/Au@ZIF‑67 electrochemical sensor**

Sara Dehdashtian ^a,^ *, Shengnian Wang ^a^, Teresa A. Murray ^a^, Mahdieh Chegeni ^b^, Sadegh Rostamnia ^c^ and Nazir Fattahi ^d^

*^a^**Institute for Micromanufacturing, Louisiana Tech University, Ruston, Louisiana 71270, United States, Center for Biomedical Engineering and Rehabilitation Sciences, Louisiana Tech University, PO Box 10157, Ruston, LA 71272, USA*

*^b^Department of Chemistry, Faculty of Science, Ayatollah Boroujerdi University, Boroujerd, 69199-69737 Iran*

*^c^Organic and Nano Group (ONG), Department of Chemistry, Iran University of Science and Technology (IUST), PO Box 16846-13114, Tehran, Iran.*

*^d^Research Center for Environmental Determinants of Health (RCEDH), Health Institute, Kermanshah University of Medical Sciences, Kermanshah, Iran*


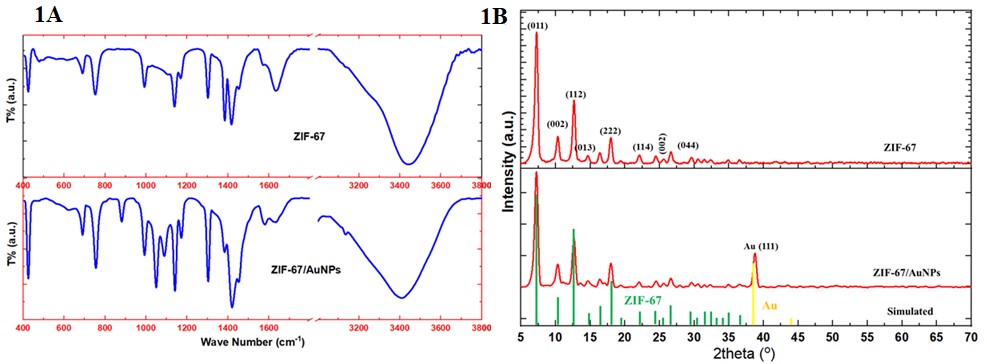


**Figure S1**. **A)** FTIR spectrum of ZIF-67 and Au@ZIF-67. **B**) XRD pattern of ZIF-67, and Au@ZIF-67.


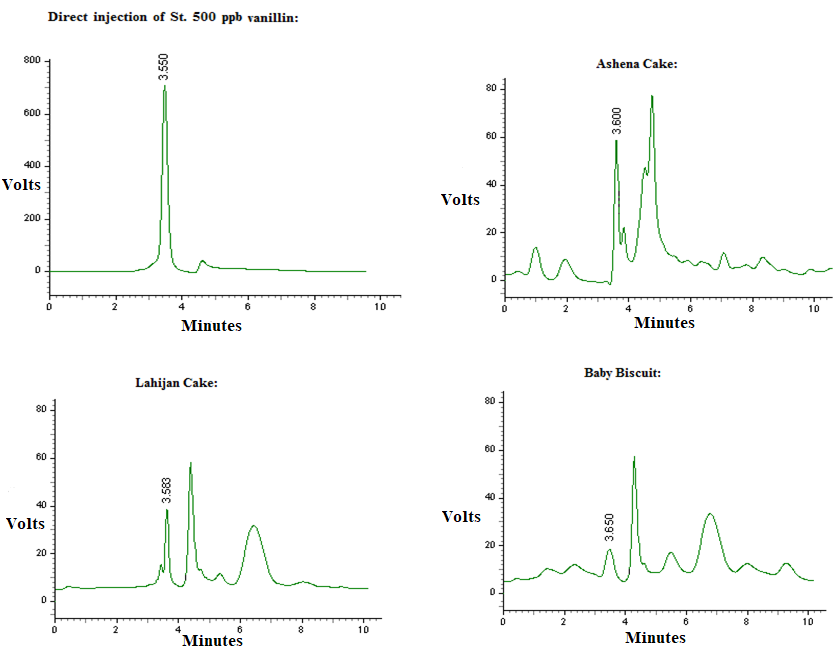


**Figure S2.** HPLC chromatograms of three real samples for determination of vanillin concentration.


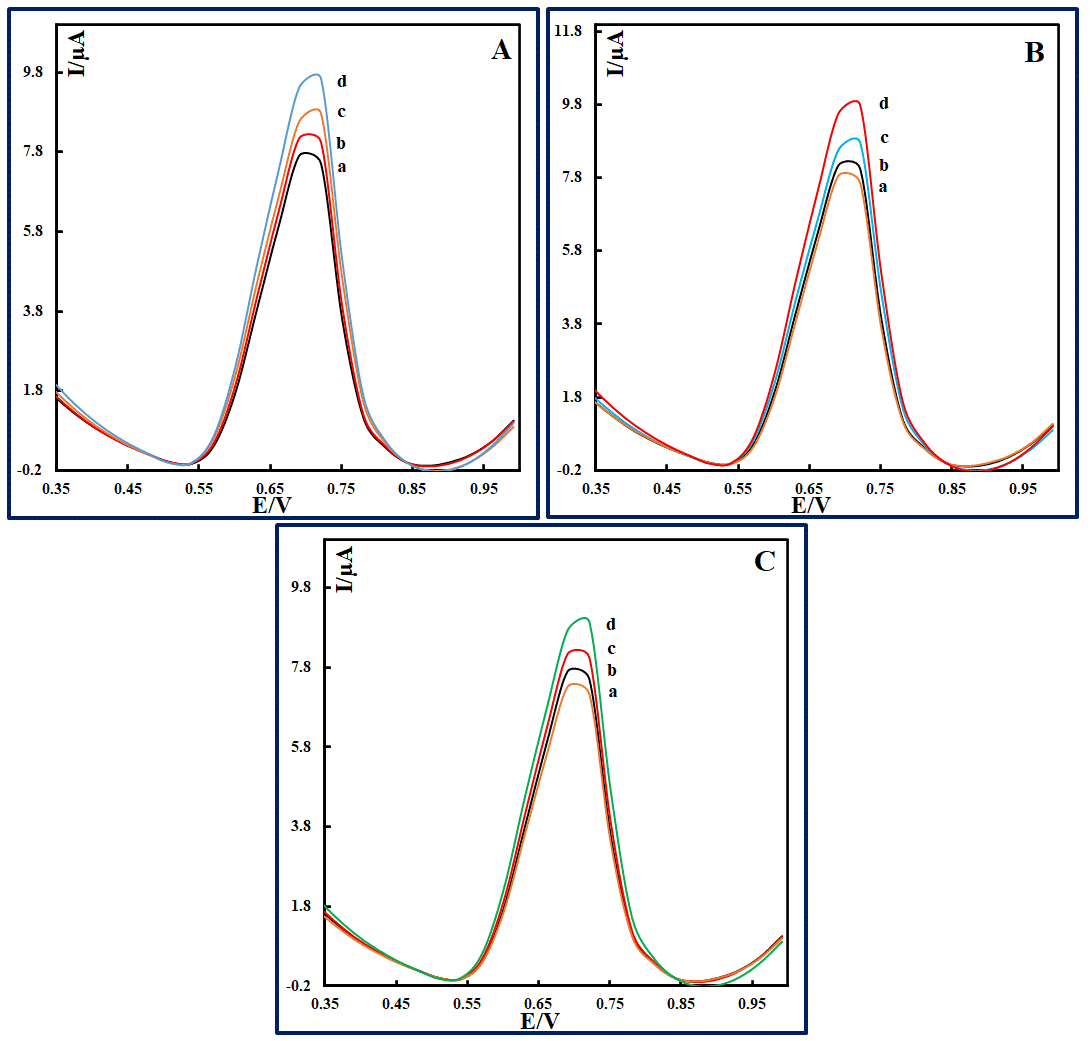


**Figure S3.** Results for the standard addition of analyte in DPV method. Panels A, B and C are Ashena cake, Lahijan cake, and Baby biscuits, respectively (a,b,c,d represent the four replicates).
